# Dysfunction of the Neurovascular Unit by Psychostimulant Drugs

**DOI:** 10.3390/ijms242015154

**Published:** 2023-10-13

**Authors:** Tam Thuy Lu Vo, Dain Shin, Eunyoung Ha, Ji Hae Seo

**Affiliations:** 1 Department of Biochemistry, Keimyung University School of Medicine, Daegu 42601, Republic of Korea; volutam@gmail.com (T.T.L.V.); eyha@dsmc.or.kr (E.H.); 2 Keimyung University School of Medicine, Daegu 42601, Republic of Korea; dain000629@naver.com

**Keywords:** drug abuse, blood–brain barrier (BBB), neurovascular unit (NVU), neurodegeneration, neuroinflammation: psychostimulant drugs

## Abstract

‘Drug abuse’ has been recognized as one of the most pressing epidemics in contemporary society. Traditional research has primarily focused on understanding how drugs induce neurotoxicity or degeneration within the central nervous system (CNS) and influence systems related to reward, motivation, and cravings. However, recent investigations have increasingly shifted their attention toward the detrimental consequences of drug abuse on the blood–brain barrier (BBB). The BBB is a structural component situated in brain vessels, responsible for separating brain tissue from external substances to maintain brain homeostasis. The BBB’s function is governed by cellular interactions involving various elements of the ‘neurovascular unit (NVU),’ such as neurons, endothelial cells, astrocytes, pericytes, and microglia. Disruption of the NVU is closely linked to serious neurodegeneration. This review provides a comprehensive overview of the harmful effects of psychostimulant drugs on the BBB, highlighting the mechanisms through which drugs can damage the NVU. Additionally, the review proposes novel therapeutic targets aimed at protecting the BBB. By understanding the intricate relationships between drug abuse, BBB integrity, and NVU function, researchers and clinicians may uncover new strategies to mitigate the damaging impact of drug abuse on brain health.

## 1. Introduction

The BBB constitutes a distinct structure within brain microvessels, characterized by the absence of fenestrae, serving as a guardian for the brain microenvironment. Its paramount role involves preventing external stimuli and chemicals from infiltrating the brain. Disruption of BBB integrity can usher in neuronal dysfunction, neurodegeneration, and neuroinflammation. The constituents of the BBB encompass brain endothelial cells, pericytes, and astrocytic endfeet. These components collaboratively create a robust defense. Each endothelial cell enveloping the capillary lumen is connected via tight junctions, while pericytes adhere to the endothelial cell surface, all enclosed by the basal lamina. Enveloping the basal lamina are astrocytic endfeet. Junctional complexes, specifically tight junctions (TJ) and adherence junctions (AJ), contribute to the BBB’s integrity. TJ is constituted by transmembrane components such as junctional adhesion molecule (JAM-1), occludin, and claudin-1, -3, -5, with claudins primarily forming the BBB structure, complemented by occludin. Adherence junctions primarily hinge on VE-cadherin, a transmembrane component that mediates cell–cell adhesion. Beyond these transmembrane proteins, cytoplasmic proteins are integral to BBB development and function. Notably, zona occludens (ZO)-1, -2, -3 (ZO-1, ZO-2, ZO-3) serve as recognition proteins for TJ placement [1,2,3].

**Figure 1:**
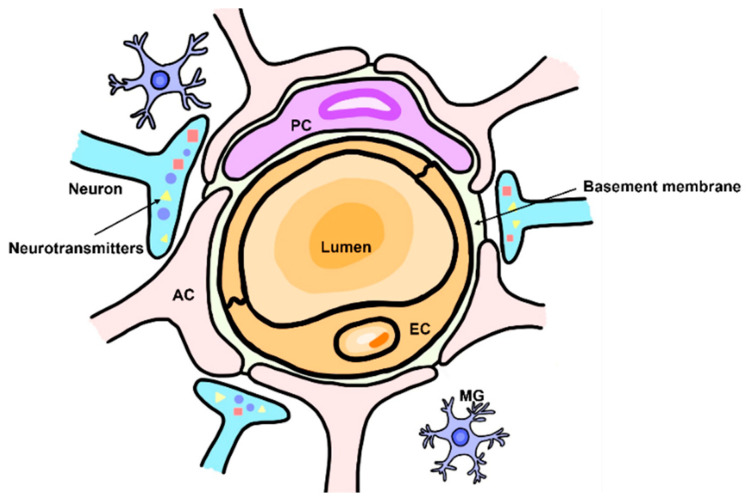
The components of neurovascular unit. Brain endothelial cell, pericyte, and astrocyte together modulate the function of BBB. Pericytes are attached to surface of endothelial cell and astrocytes surround both of them. Various types of neurons innervate to endothelial cell and astrocyte, developing neurovascular coupling together. After brain injury, microglia are activated and mediate immune responses. PC, pericyte; AC, astrocyte; EC; endothelial cell; MG; microglia.

The cellular components constituting the BBB engage in close and constant communication with neural tissue, a dynamic interaction that fosters BBB development and upholds its structural and functional integrity. This phenomenon is encapsulated within the concept of the ‘neurovascular unit (NVU),’ encompassing endothelial cells, pericytes, astrocytes, neurons, microglia, and the extracellular matrix (Figure 1) [2].

The endothelial cell, which forms the framework of the BBB, generates various factors influencing vascular tone. The extracellular matrix facilitates communication through ion diffusion, neurotransmitter exchange, and ATP transmission, contributing to cell–matrix interactions. Matrix proteins within the extracellular matrix, such as collagen, function as anchors for the endothelium and exert influence over tight junction protein expression. Pericytes intermittently line the endothelial wall, forming capillary-like structures alongside endothelial cells and astrocytes. They secrete vasoactive substances, including pericyte-derived angiopoietin, which prompts occludin expression, fortifying the BBB’s structural integrity. Pericytes and endothelial cells collaboratively establish junctional complexes like gap junctions, tight junctions, and focal adhesions. Astrocytes extend their endfeet toward brain endothelial cells, lending support to the BBB structure alongside pericytes. Additionally, studies have demonstrated that astrocytes enhance BBB characteristics through the secretion of trophic factors [1,2].

Pericytes and astrocytes foster communication not only with endothelial cells, but also with neurons. Neurons, endothelial cells, pericytes, and astrocytes together create neurovascular coupling. As glutamate is released from neuronal synapses, astrocytic calcium concentration increases, leading to the opening of K+ channels. This triggers the secretion of vasoactive substances such as neuropeptides, ATP, or nitric oxide (NO) from pericytes and astrocytic endfeet. Endothelial cells also release vasoconstrictors or vasodilators in response to diverse signals, contributing to the modulation of vascular tone. Furthermore, serotonergic, γ-aminobutyric acid (GABA)ergic, cholinergic, and noradrenergic neurons innervate microvascular endothelium and astrocytic processes [1].

Microglia, a form of neuroglial cells, represent resident macrophages spanning the brain and spinal cord, actively participating in immune responses within the CNS. When exposed to external agents like pro-inflammatory cytokines, viruses, metal ions, or neuropathological conditions such as brain trauma and ischemia, microglia release cytokines such as TNF-α, IL-β, and reactive oxygen species (ROS). These agents can down-regulate tight junction proteins and compromise BBB integrity [3].

Numerous studies have highlighted BBB damage in neuropathological conditions like ischemic stroke, drug abuse, and Alzheimer’s disease, often through diverse mechanisms. In hypoxia or ischemic stroke scenarios, expression of TJ proteins including claudin-5, occludin, and ZO-1 diminishes, and substances exacerbating BBB vulnerability, such as vascular endothelial growth factor (VEGF), NO, and ROS, are secreted by astrocytes, pericytes, and microglia [4]. Additionally, inflammation prompts the release of pro-inflammatory cytokines, chemokines, and ROS by the NVU, activating matrix metalloproteinase (MMP)-2 and -9 that can downregulate tight junction proteins and disrupt the BBB. As MMP-2 and -9 levels rise, pericytes detach from endothelial cells, and immune cells infiltrate the endothelial cell membrane [5].

Moreover, recent investigations have unveiled the breakdown of BBB in individuals abusing psychostimulants, and studies exploring BBB protection have shown positive effects on behavioral impairment. Cocaine and amphetamine-type drugs, including methamphetamine (METH) and methylenedioxymethamphetamine (MDMA), rank among the most widely used illicit psychostimulants globally [6]. Cocaine, METH, and MDMA share common characteristics such as hydrophilicity and a positive charge at pH 7.4. Consequently, these substances cannot passively cross the BBB. Nonetheless, they traverse the BBB through a carrier-mediated transport mechanism (Table 1). Cocaine and MDMA utilize membrane proton/organic cation antiporters for transport, whereas METH can utilize both membrane proton/organic cation (H+/OC) antiporters and solute carriers, including OCT2, MATE1, and MATE2-K [7]. Traditionally, psychostimulants were associated with neurotoxicity and devastation in CNS. However, recent interest has focused on the connection between BBB disruption and drug abuse. This review explores the impact of psychostimulants, particularly cocaine and amphetamine-type drugs like METH and MDMA, on BBB compromise and the various mechanisms through which they affect the components of NVU.

## 2. BBB Disruption by Drugs

Numerous studies have administered drugs such as cocaine, METH, MDMA to animals and cells to substantiate the detrimental impact of these substances on the BBB. Here, we will highlight several addictive drugs that have been shown to disrupt the BBB, both in vivo and in vitro (Table 2).

### 2.1. Cocaine

Cocaine exerts its effects by inhibiting monoamine uptake through binding to the DAT, which leads to an increase in extracellular DA and glutamate levels. Previous in vivo investigations have demonstrated that cocaine treatment elevates BBB permeability, resulting in neuronal damage and brain edema [8,11]. In a study by Jang et al., rats underwent cocaine self-administration training to examine the impact of cocaine on brain temperature and BBB integrity. This study revealed that cocaine elevated brain temperature, increased BBB permeability, and led to the release of extracellular DA and ROS [10]. Furthermore, Walker et al. reported that a group of cocaine-dependent patients exhibited lower superoxide dismutase (SOD) activity compared to a healthy group, suggesting an increased risk of BBB damage in cocaine-dependent individuals [23]. Treating brain endothelial cells with cocaine also induced BBB compromise through oxidative stress and neuroinflammation [8,9]. Additionally, Gan et al. uncovered that in a co-culture of brain microvascular endothelial cells (BMVEC) and monocytes, cocaine treatment triggered the release of pro-inflammatory cytokines (IL-6, TNF-α) in both cell types, and increased the expression of endothelial adhesion molecules like ICAM-1 and VCAM-1 in BMVEC [24].

Interestingly, cocaine-mediated neuroinflammation is closely associated with the sigma-1 receptor (S1R), a non g protein-coupled intracellular protein receptor that is widely expressed in NVU components. Cocaine binding to the σ-1 receptor induces neuroinflammation by upregulating leukocyte cell adhesion molecules in endothelial cells and pro-inflammatory cytokines in microglia. Cocaine also induces astrogliosis and neurotoxicity by binding to the S1R, which is expressed in astrocytes and neurons [25].

### 2.2. Methamphetamine (METH)

METH is a potent CNS stimulant that induces effects such as euphoria, heightened mood, hypertension, and reduced fatigue. Nevertheless, prolonged METH administration leads to significant psychiatric withdrawal symptoms upon abrupt discontinuation, including anhedonia, hypersomnia, anxiety, and intense cravings [26]. METH operates as a dopamine transporter (DAT) inhibitor, hindering the reuptake of dopamine (DA) into presynaptic terminals. This action elevates extracellular DA and glutamate levels in the synaptic cleft, resulting in damage to monoaminergic neurons, including dopaminergic and serotonergic neurons [27]. Human studies have demonstrated decreased regional cerebral blood flow (rCBF) in regions housing dopaminergic neurons among METH abusers, indicating reduced neural activity or neural damage in those areas [28]. Furthermore, a body of research underscores that both acute and chronic METH treatments significantly contribute to the disruption of the BBB [15,29].

In rodent models, high doses or prolonged METH treatment have been shown to increase brain and body temperatures, elevate BBB permeability, induce brain edema, and cause nerve cell damage. These effects are attributed to the upregulation of ROS, MMPs, and cell adhesion molecules like intercellular adhesion molecule-1 (ICAM-1) and vascular cell adhesion molecule-1 (VCAM-1) [13,15,16,17,18,30]. Moreover, in METH self-administering HIV-1 transgenic rats, the expression of TJ proteins, such as claudin-5, occludin, and ZO-1, was found to decrease. In vitro investigations have indicated that exposing brain endothelial cells to METH increased the permeability of these cells under non-toxic conditions [12,13]. METH treatment of endothelial cells led to cytoskeletal rearrangements and the redistribution of junctional proteins [12]. Furthermore, higher doses of METH were shown to increase the number of apoptotic cells by inducing endoplasmic reticulum (ER) stress [13].

### 2.3. Methylenedioxymethamphetamine (MDMA)

While low doses of MDMA are currently under evaluation in clinical trials as an adjunctive therapy for post-traumatic stress disorder (PTSD) [30] and certain other anxiety disorders [31,32], it is important to note that MDMA possesses undesired acute effects, such as arousal and insomnia. More severe effects, including hyperpyrexia, serotonin syndrome, and hyponatremia with cerebral edema, have been well-documented [33]. These effects are attributed to MDMA’s impact on the release and reuptake inhibition of serotonin and dopamine in the synaptic cleft, leading to increased binding of these neurotransmitters to noradrenergic, histaminergic, and muscarinic receptors. This, in turn, modulates mood, thermoregulation, and autonomic nervous system activity [34].

Numerous studies have demonstrated BBB dysfunction resulting from MDMA administration. An in vitro model using bovine brain microvessel endothelial cells (bBMVECs) has shown that MDMA triggers NO production and necrosis, disrupting the endothelial cell monolayer and causing BBB breakdown [19]. In vivo models have revealed that acute MDMA administration induces brain edema and increases the leakage of Evans Blue, as well as IgG immunostaining, indicating BBB permeability alterations in various brain areas such as the hippocampus, cerebellum, cortex, hypothalamus, and striatum [20,21]. The MDMA-induced BBB disruption effect is associated with a remarkable reduction in laminin and collagen-IV levels, which is caused by the increased activity of MMP9 and MMP3 [21,22].

## 3. Effects of Drugs on NVU

**Figure 2:**
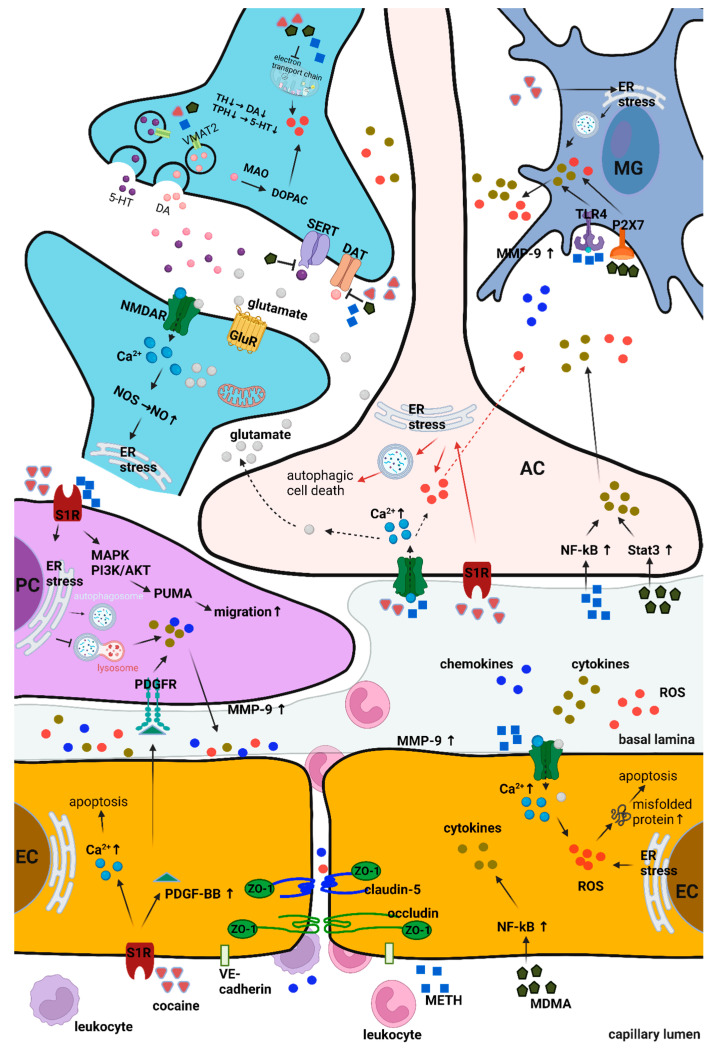
Effects of psychostimulant drugs on NVU components. Psychostimulants induce ECs to release cytokines, chemokines, and ROS by upregulating NF-κB activity, Ca^2+^ accumulation, or PDGF-BB expression, thereby elevating MMP-9 expression. Consequently, MMP-9 downregulates junctional proteins and extracellular matrix proteins in the basal lamina. Intracellular Ca^2+^ accumulation also causes apoptosis due to the production of misfolded proteins resulting from ER stress. Cocaine stimulates S1R, which activates PDGF-BB, resulting in a reduction in ZO-1 levels. METH induces the redistribution of junctional proteins from the membrane to the cytoplasm. Together, psychostimulants induce BBB disruption, further increasing infiltrated leukocytes, and provoking neuroinflammation. Subsequently, the activation of PDGFR by PDGF released from endothelial cells promotes pericytes to release cytokines and chemokines, facilitating leukocyte infiltration. While cocaine, by activating S1R, disrupts the fusion of autophagosomes and lysosomes, causing dysregulated autophagy and promoting the release of chemokines and cytokines, METH activates PUMA via activation of S1R, leading to the migration of pericytes, contributing to BBB disruption. Cocaine and METH induce the release of glutamate from activated astrocytes by promoting intracellular Ca^2+^ accumulation. Psychostimulants induce the release of ROS and cytokines from astrocytes by activating NF-κB or Stat3 signaling or through ER stress. These released cytokines and chemokines, in turn, exacerbate BBB leakage. Microglia mainly execute neuroinflammatory responses. Cytokines and chemokines released from astrocytes activate microglia. Cytokines and chemokines stimulate TLR4 on microglia, and activated microglia release pro-inflammatory cytokines, chemokines, NO, and ROS, increasing MMP-9 expression. MDMA increases P2X7 expression, leading to an increase in MMP-9 and consequent BBB leakage. Notably, cocaine, METH, and MDMA increase the release of neurotransmitters, including dopamine, serotonin, and glutamate, while inhibiting their re-uptake, resulting in an increased extracellular level of these neurotransmitters in the synaptic cleft. Intracellular psychostimulants inhibit VMAT, causing an accumulation of intracellular dopamine, which is metabolized by MAO, resulting in ROS production. Psychostimulant drugs also promote intracellular Ca^2+^ levels through excessive glutamate released from presynaptic neurons and activated astrocytes. Intracellular Ca^2+^ regulates various kinases and induces NOS activation, ER stress, neuronal cell death, and apoptosis. Additionally, psychostimulants block the mitochondrial electron transport chain, evoking ROS generation and inducing neuronal damage. Neuroinflammation and neurotoxicity are consequences of the cytokines and chemokines released from NVU cells and infiltrated leukocytes. EC, endothelial cells; AC, astrocyte; MG; microglia; PC, pericyte. The figure was created with Biorender.com on 30 September 2023 (agreement number for publication: EB25WVUPA7).

Each of these drugs exerts deleterious effects on the components of the NVU through diverse mechanisms, culminating in BBB dysfunction (Figure 2). This section will elucidate the impacts of these drugs on NVU cells and delve into the underlying mechanisms responsible for these effects.

### 3.1. Neuron

Drugs exert their detrimental effects primarily on neurons, culminating in neurodegeneration and the subsequent disruption of the BBB. Notably, cocaine, METH, and MDMA interfere with monoamine transporters.

Cocaine blocks DAT and prompts the vesicular monoamine transporter (VMAT)-2 to preferentially package catecholamines, leading to an elevation in synaptic DA levels [35]. Similarly, METH hinders the functioning of both the DAT and VMAT-2 by inducing DAT internalization and disrupting the VMAT proton gradient. At higher doses, METH redistributes VMAT as well [36]. Like other amphetamines, MDMA enhances extracellular levels of monoamines, including DA, serotonin (5-HT), norepinephrine (NE), by stimulating monoamine release and inhibiting their re-uptake by interacting with their respective transporters (DAT, SERT, NET). Notably, MDMA exhibits a higher affinity in ligand binding for SERT compared to DA, therefore, induces higher level of 5-HT than DA. MDMA also blocks VMAT2, further enhancing monoamine release [37].

By obstructing monoamine transporters, these drugs elevate extracellular DA levels. Cocaine-mediated surplus DA generates excessive ROS during DA metabolism, particularly monoamine oxidase (MAO) metabolism by auto-oxidation or MAO-catalyzed DA. The oxidative conversion of DA by MAO yields ROS, in particular hydrogen peroxide (H_2_O_2_) and 3,4-dihydroxyphenylacetic acid (DOPAC), a DA metabolite. The oxidative stress stemming from the excessive ROS produced during DA metabolism ultimately leads to neuronal cell death. Moreover, DA undergoes auto-oxidation during metabolism, resulting in the production of neurotoxic compounds like superoxide, toxic peroxynitrite, and 6-hydroxydopamine, which trigger axon degeneration [35]. In addition to DA metabolism-generated ROS production, METH treatment generates ROS by downregulating iron chelators, leading to increased free ions—source of ROS. Furthermore, high levels of cytochrome P450s, used to detoxify METH, can also generate ROS, leading neuronal cell death. Another mechanism of METH-induced ROS production is that METH exposure triggers mitochondria dysfunction, resulting in ROS production, inducing apoptosis [38]. Likewise, ROS production increases following MDMA exposure-promoted neurotoxicity through inhibiting mitochondria electron transport chain. Metabolites of MDMA is also a cause of ROS generation as its metabolites can undergo oxidation to quinolones, which produce semiquinones radicals [39].

In tandem with DA, these psychostimulant drugs also elevate extracellular glutamate levels. This enhanced glutamate stimulates N-methyl-D-aspartate receptors (NMDARs) and α-amino-3-hydroxy-5-methyl-4-isoxazolepropionic acid (AMPA)/kainate (KA) receptors, inducing calcium (Ca^2+^) influx alongside glutamate. Ordinarily, extracellular Ca^2+^ levels far exceed intracellular levels, and the entry of Ca^2+^ into cells governs various signaling pathways. The Ca^2+^ influx initiated by N-methyl-D-aspartate (NMDA) and AMPA/KA receptors activates diverse kinases and nitric oxide synthase (NOS), which produces NO. This cascade induces endoplasmic reticulum (ER) stress, neuronal cell death, apoptosis, and, ultimately, damage to monoaminergic neurons, such as dopaminergic and serotonergic neurons [40,41].

Furthermore, chronic METH consumption depresses tryptophan hydroxylase (TPH) in the neostriatum and hippocampus, as well as tyrosine hydroxylase (TH) in the neostriatum. TPH and TH are pivotal enzymes responsible for creating serotonin and dopamine, respectively. Consequently, METH abuse leads to a reduction in the number of dopaminergic and serotonergic neurons [42].

### 3.2. Endothelial Cell and Extracellular Matrix (ECM)

The mechanisms underlying the breakdown of endothelial cells induced by drugs can be largely attributed to the degradation of junctional proteins via the activation of MMPs and cytoskeletal rearrangement.

MMPs, a group of proteases belonging to the extracellular zinc endopeptidases family, play a significant role in this context, with MMP-2 and -9 being particularly relevant. Following brain injury, especially after drug exposure, these MMPs are dysregulated, causing damage to endothelial cells and the extracellular matrix. MMPs are exacerbated by factors like free radicals, inflammatory cytokines, and vascular adhesion molecules. Drug exposure triggers the secretion of these stimulants, leading to the degradation of proteins in both the endothelial cell and extracellular matrix [43]. METH, for instance, prompts the release of glutamate in synapses, leading to ROS generation through metabolic stimulation and NMDA-mediated Ca^2+^ influx. These ROS then disrupt TJ proteins by increasing MMP-9 levels. Additionally, glutamate causes alterations in occludin distribution and expression [44]. In the case of treating METH in HIV-1 transgenic rats, lipopolysaccharide (LPS) invades the hippocampus, binds to toll-like receptor 4 (TLR4), and triggers immune responses such as cytokine release and leukocyte infiltration, resulting in elevated MMP levels [14]. METH treatment also elevates MMP-9 levels, contributing to the degradation of ECM. Simultaneously, METH increases monocyte adhesion to microvascular endothelial cells, thereby facilitating monocyte transmigration and promoting the intrusion of HIV-infected cells into CNS [45]. Similarly, MDMA induces an increase in IL-1β release, likely through NF-κB activation, subsequently activating MMP-9 activity, which in turn triggers a decrease in claudin-5 and the degradation of ECM proteins such as laminin and collagen IV [21,22]. In a similar fashion, cocaine treatment activates prolidase, which cleaves accumulated proline from imidodipeptides and imidotripeptides due to MMP activity, resulting in ECM degradation [9].

Furthermore, psychostimulants induce BBB permeability through the rearrangement of endothelial cell cytoskeletons and the redistribution of junctional proteins. Cocaine has been found to reduce the ZO-1 protein level through PDGF-BB signaling in both in vivo and in vitro studies. Upon activation of S1R in endothelial cells following cocaine treatment, PDGF-BB activation increases, leading to a decrease in ZO-1 protein levels [8]. METH disrupts cytoskeletal morphology not only by elevating F-actin levels but also by provoking stress fiber formation. Additionally, METH induces the redistribution of ZO-1 and VE-cadherin from the membrane to the cytoplasm [12].

Moreover, some findings indicate that METH triggers ER stress in brain endothelial cells. The increased ROS caused by METH disrupts protein folding, leading to an accumulation of misfolded proteins, subsequently inducing ER stress, apoptosis, and neuroinflammation [13].

### 3.3. Astrocyte

Several psychostimulants induce a phenomenon known as ‘astrogliosis’ or ‘astrocytosis,’ characterized by the amplification of reactive astrocytes that secrete various molecules capable of disrupting the BBB. Upon activation, these astrocytes release proinflammatory cytokines such as TNF-α, IL-1B, and IL-6. This activation triggers microglial activation, an inflammatory response, the death of dopaminergic neurons, and oxidative stress during neurodegenerative processes [2,5].Psychostimulants promote astrocyte activation by elevating levels of ROS, inducing Ca^2+^ influx, hyperthermia, and ER stress, among other factors. Cocaine and METH, for example, increase ROS production and intracellular Ca^2+^ levels in astrocytes through the release of hydrogen peroxide and lipid peroxidation following the increase in dopamine levels [46]. METH leads to an expansion of the reactive astrocyte population by inducing hyperthermia [15]. As demonstrated by Periyasamy et al., cocaine treatment induces astrocytosis through ER stress-mediated autophagy [47].

Astrogliosis induced by MDMA produces contrasting results. While several studies report that MDMA treatment does not trigger astroglial activation [48,49,50,51], numerous others provide evidence that MDMA treatment increases glial fibrillary acidic protein (GFAP) immunoreactivity, a marker of astrogliosis, in response to neuronal damage [52,53,54,55,56]. This discrepancy may arise from differences in rodent models as well as brain regions studied. Recently, it is evidenced that effect of MDMA on astrocyte activation may involve increased STAT3 signaling, which is a modulator of astrocyte differentiation, leading to nigrostriatal dopamine neuron degeneration [57]. However, an investigation by Mercedes Pérez et al. proposed that MDMA-promoted astrocytic activation may be a BBB-independent, since GFAP remained increased though treatment of SB-3CT inhibited MMP-9-induced BBB disruption [22]. Interestingly, MDMA affects astrocyte function in different sexes differently. This may due to different serotonin levels in sex differences [58].

### 3.4. Pericyte

Pericytes are the most abundant cell type among NVU cell types in the CNS and the only cell that directly contacts the BBB endothelium. Interestingly, pericytes are still the least characterized due to the lack of pan-pericyte specific markers since their origins vary from the different brain regions [59,60]. Numerous studies have highlighted the significant role of pericytes as mediators of neuroinflammation. Pericytes, directly attached to endothelial cells, initiate inflammatory responses against external stimuli, including drugs [1]. Despite the importance of pericytes in the regulation of BBB integrity, rCBF, and transcytosis of endothelial cells, the specific mechanisms by which psychostimulants damage pericytes, causing BBB breakdown, remain elusive. Recently, researchers have consistently observed that pericytes display active inflammatory responses when exposed to psychostimulant drugs.

METH has been reported to upregulate expression of p53 up-regulated modulator of apoptosis (PUMA) in pericytes via sigma-1 receptor, thereby promoting pericyte migration from the endothelial basement membrane, leading to BBB damage [61]. However, up to today, effects of an MDMA-another amphetamine-type drug, like METH, on pericytes are yet to be discovered.

In the study conducted by Sil et al., the administration of cocaine to both mice and primary human brain vascular pericytes (HBVPs) resulted in ER stress-induced dysregulated autophagy, thereby generating the secretion of pro-inflammatory cytokines, setting off a cascade of neuroinflammatory processes [62]. Additionally, exposure of pericytes to cocaine led to the release of the chemokine CXCL10, which in turn induced the transmigration of monocytes [63].

### 3.5. Microglia

Microglia, as immune effector cells within CNS, primarily drive neuroinflammation. In response to psychostimulants, other components of NVU, such as endothelial cells and astrocytes, release pro-inflammatory cytokines and chemokines that attract microglia and monocytes [3]. Cocaine, for example, increases the expression of adhesion molecules like ICAM-1 and VCAM-1 in endothelial cells, leading to leukocytic infiltration through the BBB [29]. METH activates microglia by inducing the release of pro-inflammatory mediators via the Toll-like receptor 4 (TLR4) pathway.

Psychostimulants can also directly impact microglia by inducing the release of pro-inflammatory mediators and triggering cell death. Cocaine, through ER stress-mediated autophagy, prompts the release of cytokines in microglia, endothelial cells, and astrocytes [64]. A line of evidence proves that MDMA likely exerts neurotoxicity through microglia activation than astroglia [58,65]. MDMA administration increases expression of the ionotropic purinergic receptor P2X7 or adenoside receptor A2a in activated microglia, following by release of proinflammatory prostaglandins, cytokines and free radical formation, which contribute to neuroinflammation and neurotoxicity [21,66,67,68].

Furthermore, a connection has been established between drug abuse and the spread of HIV. HIV viral proteins and drugs collectively trigger an inflammatory response in the CNS, leading to the disruption of BBB integrity. Drugs can exacerbate HIV transmission and replication. Cocaine and METH initiate the transcriptional initiation of HIV genes that are crucial for the replication of viral proteins [69]. Cocaine also promotes the infiltration of HIV-1 viral proteins into brain tissue, while METH, in conjunction with the HIV viral protein gp120, reduces the expression of claudin-3, -5, ZO-1, and occludin [29,70].

## 4. Targeting Strategy

While drugs contribute to BBB breakdown and damage to NVU cells through various mechanisms, they share common targets for modulation. ROS serves as a representative example generated by all drug types, inducing neurotoxicity, neuroinflammation, ER stress, and more. Consequently, numerous studies have indicated that treating drugs with ROS-scavenging molecules can alleviate BBB breakdown. For instance, treating with 4-Hydroxy-TEMPO (TEMPOL) mitigated the dopamine-blocking activity of cocaine. Phenyl n-tert-butyl nitrone (PBN) treatment with METH showed a reduction in the redistribution of junctional proteins and the formation of F-actin in HBMECs [10,12]. The ROS scavenger NBP (DL-3-n-butylphthalide) and the NOX inhibitor apocynin attenuated METH-induced ER stress response [13].

ER stress arises from the release of ROS and cytokines prompted by drugs, leading to cellular apoptosis. METH treatment increased the expression of ER stress markers (IRE1, PERK, ATF6) and the ER stress mediating transcription factor C/EBP Homologous Protein (CHOP). Co-treating the ER stress inhibitor 4-phenylbutyric acid (4-PBA) with METH prevented changes in occludin and claudin-5 expression, as well as BBB permeability. Knockdown of CHOP increased cell viability and decreased the number of apoptotic cells. CHOP depletion also alleviated the decrease in cellular integrity and TJps in endothelial cells [13].

Melatonin, an endogenous ligand synthesized in the pineal gland, regulates timekeeping functions, circadian maintenance, and also acts as an antioxidant and anti-inflammatory mediator. Co-treatment of melatonin with METH restored junctional protein levels, decreased BBB leakage and cytokine release. Melatonin also increased the levels of antioxidant NAD(P)H quinone oxidoreductase 1 (NQO1) and Nrf2, a representative transcription factor of antioxidant genes [18].

As elucidated above, psychostimulants drugs activate various transcription factors and enzymes at the transcriptional or translational level. Therefore, it appears feasible to inhibit drug effects on the BBB by transfecting siRNA or silencing micro-RNA. Bai et al. demonstrated that METH up-regulated mi-R143 after activating S1R, and mi-R143 led to a decrease in claudin-5, occludin, and ZO-1 levels. miR-143 targeted the p53 unregulated modulator of apoptosis (PUMA) to activate NF-κB and p53, and depletion of p53 and NF-κB recovered the expression level of claudin-5 and occludin [71].

## 5. Conclusions

Psychostimulant drugs like METH, MDMA, and cocaine can damage the BBB, inducing brain edema and neurotoxicity. Apart from acute cognitive impairment, damage to the BBB and NVU could promote more serious CNS disorders such as stroke, Parkinson’s disease, and Alzheimer’s disease. Therefore, considerable effort has been invested in investigating the significant mechanisms through which each drug exerts destructive effects on the NVU. Despite the diversity of drug-mediated molecular mechanisms, drugs commonly induce oxidative stress, neuroinflammation, Ca^2+^ influx, and cell apoptosis by excessive extracellular dopamine and glutamate. For this reason, methods to reduce extracellular monoamine levels or inhibit neuroinflammation and release of ROS would be required. Currently, materials for reducing the breakdown of BBB, such as ROS scavengers, ER stress inhibitors, and MLCK inhibitors, were used in many in vivo and in vitro studies. Those materials would also have valuable effects on alleviating brain dysfunctions in drug abusers. Further studies should be conducted to investigate the molecular mechanisms underlying the positive effects of novel materials, such as melatonin, 5-HT inhibitor, and S1P, on the breakdown of the BBB caused by drug abuse. Finally, the adverse effects of the therapeutic methods should be studied in various aspects to facilitate the development of more refined approaches.

## Figures and Tables

**Table 1:** Structure and characteristics of widely used illicit psychostimulants.

Substance	Structure	Hydrophilic (LogD < 2)	% Positively Charged at pH 7.4	Transporter
Cocaine	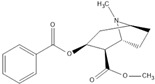	0.82	96.5%	H^+^/OC antiporter
METH	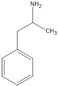	−0.44	99.9%	H^+^/OC antiporter, OCT2, MATE1, and MATE2-K
MDMA	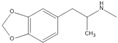	−0.76	99.8%	H^+^/OC antiporter

**Table 2:** The destructive effects of addictive psychostimulants on the BBB.

Substance	Model	Animal/Cell	Effect	Treating Time
Cocaine	In vitro	HBMEC ^1^	BBB permeability ↑ leukocyte adhesion ↑ cytokines level ↑ junctional proteins level ↓	24 h [8,9]
	In vivo	rat self-administration	oxidative stress ↑ DA release ↑	7 days [10]
			
		rat, mouse injection	BBB permeability ↑ interstitial edema temperature ↑	7 days [8] 90 days [11]
METH	In vitro	HBMEC ^1^	oxidative stress ↑ BBB permeability ↑ electrical resistance ↓ cytoskeletal rearrangement redistribution of junctional proteins	24 h [12]
			
		bEnd. 3 cell	apoptosis electrical resistance ↓ claudin-5, occludin level ↓	72 h [13]
			
	In vivo	rat self-administration	BBB permeability↑ MMP-9 level↑ junctional proteins level ↓	21 days [14]
			
		rat, mouse injection	brain, muscle temperature ↑ BBB permeability ↑ perineuronal edema nerve cell damage cell adhesion molecules ↑ oxidative stress ↑	80 min [15] 4 h [16] 1 day [13] 3 days [17] 11 days [18]
MDMA	In vitro	bBMVECs ^2^	necrosis junctional proteins level ↓	24 h [19]
	In vivo	rat, mouse injection	temperature ↑ BBB leakage ↑ perineuronal edema nerve cell damage	4 h [20]
		rat injection	temperature ↑ BBB leakage ↑ nerve cell damage MMP-9, MMP3 activity ↑ perineuronal edema claudin-5 level ↓	24 h [21,22]

^1^ human brain microvascular endothelial cell, ^2^ bovine brain microvascular endothelial cell, ↑ increase, ↓ decrease.
